# Circadian Regulation of IOP Rhythm by Dual Pathways of Glucocorticoids and the Sympathetic Nervous System

**DOI:** 10.1167/iovs.61.3.26

**Published:** 2020-03-17

**Authors:** Keisuke Ikegami, Yasufumi Shigeyoshi, Satoru Masubuchi

**Affiliations:** 1 Department of Physiology, School of Medicine, Aichi Medical University, Nagakute, Aichi, Japan; 2 Department of Anatomy and Neurobiology, Faculty of Medicine, Kindai University, Osaka-Sayama, Osaka, Japan

**Keywords:** circadian clock, adrenal glucocorticoid, intraocular pressure, sympathetic nerve system, suprachiasmatic nucleus

## Abstract

**Purpose:**

Elevated IOP can cause the development of glaucoma. The circadian rhythm of IOP depends on the dynamics of the aqueous humor and is synchronized with the circadian rhythm pacemaker, that is, the suprachiasmatic nucleus. The suprachiasmatic nucleus resets peripheral clocks via sympathetic nerves or adrenal glucocorticoids. However, the detailed mechanisms underlying IOP rhythmicity remain unclear. The purpose of this study was to verify this regulatory pathway.

**Methods:**

Adrenalectomy and/or superior cervical ganglionectomy were performed in C57BL/6J mice. Their IOP rhythms were measured under light/dark cycle and constant dark conditions. Ocular administration of corticosterone or norepinephrine was also performed. Localization of adrenergic receptors, glucocorticoid receptors, and clock proteins Bmal1 and Per1 were analyzed using immunohistochemistry. Period2::luciferase rhythms in the cultured iris/ciliary bodies of adrenalectomized and/or superior cervical ganglionectomized mice were monitored to evaluate the effect of the procedures on the local clock. The IOP rhythm of retina and ciliary epithelium-specific *Bmal1* knockout mice were measured to determine the significance of the local clock.

**Results:**

Adrenalectomy and superior cervical ganglionectomy disrupted IOP rhythms and the circadian clock in the iris/ciliary body cultures. Instillation of corticosterone and norepinephrine restored the IOP rhythm. β2-Adrenergic receptors, glucocorticoid receptors, and clock proteins were strongly expressed within the nonpigmented epithelia of the ciliary body. However, tissue-specific *Bmal1* knock-out mice maintained their IOP rhythm.

**Conclusions:**

These findings suggest direct driving of the IOP rhythm by the suprachiasmatic nucleus, via the dual corticosterone and norepinephrine pathway, but not the ciliary clock, which may be useful for chronotherapy of glaucoma.

Circadian clocks are highly conserved in all living organisms and regulate (approximately) 24-hour (circadian) rhythms in multiple physiologic and behavioral processes, including sleep–wake cycles, endocrine systems, and metabolism. The suprachiasmatic nucleus (SCN), a paired structure in the anterior hypothalamus located above the optic chiasma, acts as a circadian pacemaker in mammals. Circadian rhythm is generated by a transcription/translation feedback loop, which regulates the expression of clock genes.^1^ The circadian locomotor output cycles kaput (Clock) protein, and brain and muscle Arnt-like protein 1 (Bmal1) heterodimerize to form a transcriptional activator complex to activate the *Period* (*Per*) and *Cryptochrome* (*Cry*) repressor genes. Moreover, the SCN receives light information from the retina through the retinohypothalamic tract and governs daily rhythms throughout the body.^2^ Most peripheral tissues and cells are also synchronized with the SCN and mediated through various pathways mainly involving the autonomic nervous system and endocrine signals.^3^ However, the entrainment mechanism has not been fully elucidated, owing to its complexity.

Sympathetic pathway transmits circadian information to the ciliary body of the eye, via the superior cervical ganglion (SCG), to regulate pupil size, and to the pineal gland, to regulate melatonin synthesis.^4^ Glucocorticoids such as corticosterone (CORT), secreted from the adrenal glands are strong humoral entrainment factors for many organs, and are regulated by the SCN via the hypothalamus–pituitary–adrenal axis.^5^

IOP also has a circadian rhythm with nocturnal acrophase, which is determined by the balance between aqueous humor production by the ciliary body epithelium and its drainage through the trabecular and uveoscleral outflow. In humans, the IOP rhythm is affected by postural changes. IOP increases significantly in a supine compared with the seated position; however, nocturnal IOP increases irrespective of posture.^6^ IOP is elevated at night in nocturnal rats, mice, and rabbits, as well as in diurnal humans.^7^ Therefore, the results from animal models of circadian IOP rhythm are thought to be applicable to humans.

Glaucoma is the most common cause of irreversible blindness, and yet no effective cure exists. Elevated IOP contributes to glaucoma development and progression characterized by vision loss.^8^ Nocturnal IOP is also elevated in patients with glaucoma,^6^^,^^9^ and the IOP rhythm undergoes phase shifts in those with POAG and normal-tension glaucoma.^9^ IOP rhythm is also disrupted in night shift workers.^10^ A disrupted circadian IOP rhythm could increase the risk of glaucoma.^11^ Thus, regulation of nocturnal IOP is central in glaucoma management.

IOP rhythm is thought to be controlled by the SCN^12^ and has been reported to be mediated by sympathetic nerve-released norepinephrine (NE).^13^^–^^15^ However, individuals with Horner syndrome, with unilateral reduced or absent sympathetic innervation, show normal circadian aqueous flow patterns.^16^ Although adrenergic β1/2 receptors mainly mediate sympathetic nerve regulation of IOP, mice with these receptors knocked out still maintain IOP rhythm.^7^ Furthermore, many peripheral clocks in rodents are controlled by CORT.^17^ Although adrenalectomy (ADX) reportedly dampens IOP rhythms in mice under light/dark cycle,^18^ the circadian aqueous flow pattern is normal in human patients after surgical ADX.^19^ Therefore, sympathetic nerves and adrenal glucocorticoids alone cannot fully explain the mechanism of IOP rhythmicity.

Both glucocorticoids and sympathetic nerves act as entrainment factors in some systems^20^ and interactions between glucocorticoids and sympathetic nervous system have been reported.^21^^,^^22^ Hence, this study aimed to evaluate IOP rhythmicity and identify the pathway of regulation by the SCN after surgical removal of adrenal glands and SCG in mice, followed by instillating NE and CORT. Furthermore, the role of clock function in IOP rhythm generation of ciliary body and retina was assessed using ciliary epithelium-specific *Bmal1* knockout mice.

## Methods

### Animals

Five-week-old male C57BL/6J and Balb/c mice (*N* = 130; Japan SLC Inc., Shizuoka, Japan) were purchased and housed in plastic cages (170 wide × 240 deep × 125 high mm, Clea, Tokyo, Japan) under a 12-hour light (200 lux)/12-hour dark cycle (12L12D, 0800 light ON, 2000 light OFF) under constant temperature (23 ± 1°C). For real-time bioluminescence recording, Period 2::Luciferase fusion protein (Per2::Luc) knock-in mice^23^ were provided by Dr. Joseph Takahashi (Southwestern Medical Center, Texas University, TX). Food (CE-2; CLEA) and water were provided ad libitum. All animal experiments were approved by the Committee of Animal Care and Use of Aichi Medical University and Kindai University. All experimental procedures were conducted in accordance with institutional guidelines for the use of experimental animals and the ARVO Statement for the Use of Animals in Ophthalmic and Vision Research.

### ADX and Superior Cervical Ganglionectomy (SCGX)

SCGX and ADX were performed in 5-week-old C57BL/6J mice and Per2::Luc heterozygous mice under general anesthesia, as previously reported.^17^^,^^24^ Because ADX causes mineralocorticoid deficiency, ADX mice were given 0.9% saline ad libitum instead of water after surgery. IOP measurements were started 2 weeks after surgery to allow for the IOP rhythm to disrupt.

### IOP Measurement

IOP measurements were performed with a tonometer (Icare TonoLab, TV02; Icare Finland Oy, Espmoo, Finland), as previously reported.^12^ All mice were kept under 12L12D conditions for more than 2 weeks before taking IOP measurements and then transferred to constant dark (DD) conditions. Unanesthetized mice were gently held using a sponge. The IOP rhythm was obtained by measuring IOP at 4-hour intervals under 12L12D conditions and on the first day of DD; at zeitgeber time 2 (1000), 6 (1400), 10 (1800), 14 (2200), 18 (0200), and 22 (0600); and circadian time (CT) 2 (1000), 6 (1400), 10 (1800), 14 (2200), 18 (0200), and 22 (0600), respectively. The time of light ON defines as zeitgeber time 0 (0800). CT was based on the internal free-running period. By convention, CT0 is the beginning of the subjective day and CT12 is the beginning of the subjective night. CT12 (2000) defined the onset of activity in mice. IOPs were measured during the light phase under light (200 lux) conditions and during the dark phase under dim red-light conditions. At each time point, four IOP measurements (five per eye, first measurement was discarded to exclude handling-induced high IOP) were averaged and used for further analysis of rhythmicity and statistics.

### Analysis of Rhythmicity

When statistically significant differences in IOP rhythm were observed by one-way ANOVA, cosinor curve fitting was performed in Graph Pad Prism 6 (GraphPad Software Inc., San Diego, CA) as previously described.^25^ Temporal profiles with values of R^2^ that were greater than 0.2 were classified as rhythmic.

### Drug Treatment

A single drop (30 µL) of NE solution (1 mg/mL; # 081-104758, Daiichi-Sankyo, Tokyo, Japan) and CORT (1 mg/mL saline; C0388, TCI, Tokyo, Japan) was instilled onto bilateral eyes during CT0 to CT1 or CT12 to CT13 on the first day of DD in 8-week-old male sham and ADX+SCGX mice. During instillation, mice were gently restrained by their necks held back, under dim red-light conditions.

### Immunohistochemistry

Paraffin sections (5 µm) of paraformaldehyde-fixed eyes of albino Balb/c mice and frozen sections (14 µm) of paraformaldehyde-fixed eyes of C57BL/6J mice were used for immunohistochemistry.^25^ We used rabbit polyclonal antibody against Adrb2 (1:2000, bs-0947, Bioss), glucocorticoid receptor (GR) (1:200, sc-1004, Santa Cruz Biotechnology, Dallas, TX), guinea pig polyclonal antibody against Per1 (1:200, PM091, MBL, Nagoya, Japan) and mouse monoclonal antibody against Bmal1 (1:1000, sc-365645, Santa Cruz Biotechnology, Dallas, TX), at 4°C for 48 hours. This was followed by incubation with biotinylated goat antirabbit IgG (120 minutes; 1:1000; PK-6101, Vector Laboratories, Burlingame, CA), and then avidin–biotin complex solution (120 minutes; 1:400; Vectastain Elite ABC Kit, Vector Laboratories) before substrate visualization using 3,3′-diaminobenzidine (349-00903, Dojindo, Kumamoto, Japan). For immunofluorescence, sections were incubated with donkey polyclonal AlexaFluor 488-conjugated antirabbit IgG (1:400; R37118, Thermo Fisher Scientific, Waltham, MA) and goat polyclonal DyLight 549-conjugated antiguinea pig IgG (1:400; 606-142-129, Rockland Immunochemicals Inc, Pottstown, PA) secondary antibody for 120 minutes (1:400; R37118, Thermo Fisher Scientific).

### Real-Time Bioluminescence Recording and Analysis of Circadian Rhythm

We performed real-time bioluminescence recording of circadian rhythms as previously reported.^26^ Briefly, brains and ciliary body/iris complex of adult heterozygous Per2::Luc fusion reporter mice were quickly removed under general anesthesia and immersed in ice-cold Hank's buffered saline (09735-75, Nacalai tesque, Kyoto, Japan). The hypothalamus, containing the central part of SCN was frontally sectioned at 200 µm. Then, the bilateral SCN and iris/ciliary body were placed on a culture membrane (Millicell-CM PICM0RG50, Merck Millipore, Darmstadt, Germany) in a 35-mm petri dish and cultured at 37°C with 1.2 mL of medium containing Dulbecco's modified Eagle medium (Gibco, Carlsbad, CA), B-27 supplement (Gibco), 100 U/mL penicillin, and 100 µg/mL streptomycin, and were then transferred to the culture medium containing D-luciferin potassium salt (200 µM, Wako, Tokyo, Japan) and sealed using parafilm to prevent evaporation. Luminescence emission of the whole explant was examined using a photomultiplier tube (AB-2550, Kronos Dio, ATTO, Tokyo, Japan) for 1 minute at 10-minute intervals. Data analyses were performed using the Kronos Data Analysis Software (ATTO), Clock Lab (ver. 6) (Actimetrics, Wilmette, IL), and Microsoft Excel. Iris/ciliary body cultures underwent medium changes at 7- to 8-day intervals.

For analysis of CORT-induced Per2 expression, iris/ciliary body cultures were treated with the GR agonist dexamethasone (DEX) (100 nM; TCI, D1961) at medium change. After 30 minutes, these agents were washed out.

### Generation of Conditional *Bmal1* Knockout Mice

To generate Chx10-*Bmal1* knockout mice, Chx10-Cre mice were mated to mice with a conditional *Bmal1* allele (*Bmal1^flox/flox^*) (*N* > 9 backcrossed to C57BL/6J, Jackson Laboratory, Bar Harbor, ME, #007668).^27^ *Bmal1^flox/flox^* mice were provided by Michihiro Mieda (Kanazawa University, Ishikawa, Japan). We purchased the sperm of Chx10-Cre (B6.Cg-Tg [Chx10-cre] G5 Tfur/TfurRbrc mice) mice from RIKEN BioResource Research Center (Tsukuba, Japan) that were generated in Takahisa Furukawa (Osaka University).^28^ Genotyping was performed using multiplex PCR and primers; for *Bmal1*-flox, 5′-ACTGGAAGTAACTTTATCAAACTG-3′ (forward L1), 5′-CTGACCAACTTGCTAACAATTA-3′ (forward L2), and 5′-CTCCTAACTTGGTTTTTGTCTGT-3′ (reverse R4); for Chx10-Cre, 5′-GTCTCCTAGCCTTTGCGTTCAGAC-3′ (forward), and 5′-TTCGGCTATACGTAACAGGG-3′ (reverse).

### Statistical Analysis

All data are shown as mean ± SEM. Statistical comparisons were made using GraphPad Prism 6 (GraphPad Software Inc., San Diego, CA) or Excel-Toukei 2012 software (Social Survey Research Information Co. Ltd., Osaka, Japan). Student's *t*-tests were used to compare two groups and one-way ANOVA with Tukey's multiple comparison test for more than two groups. Time and group interactions or genotype and group interactions were compared by two-way ANOVA with Tukey's multiple comparison test. Differences with a *P* values of less than 0.05 were considered statistically significant.

## Results

### ADX and SCGX Attenuate IOP Rhythm

**Figure 1.**
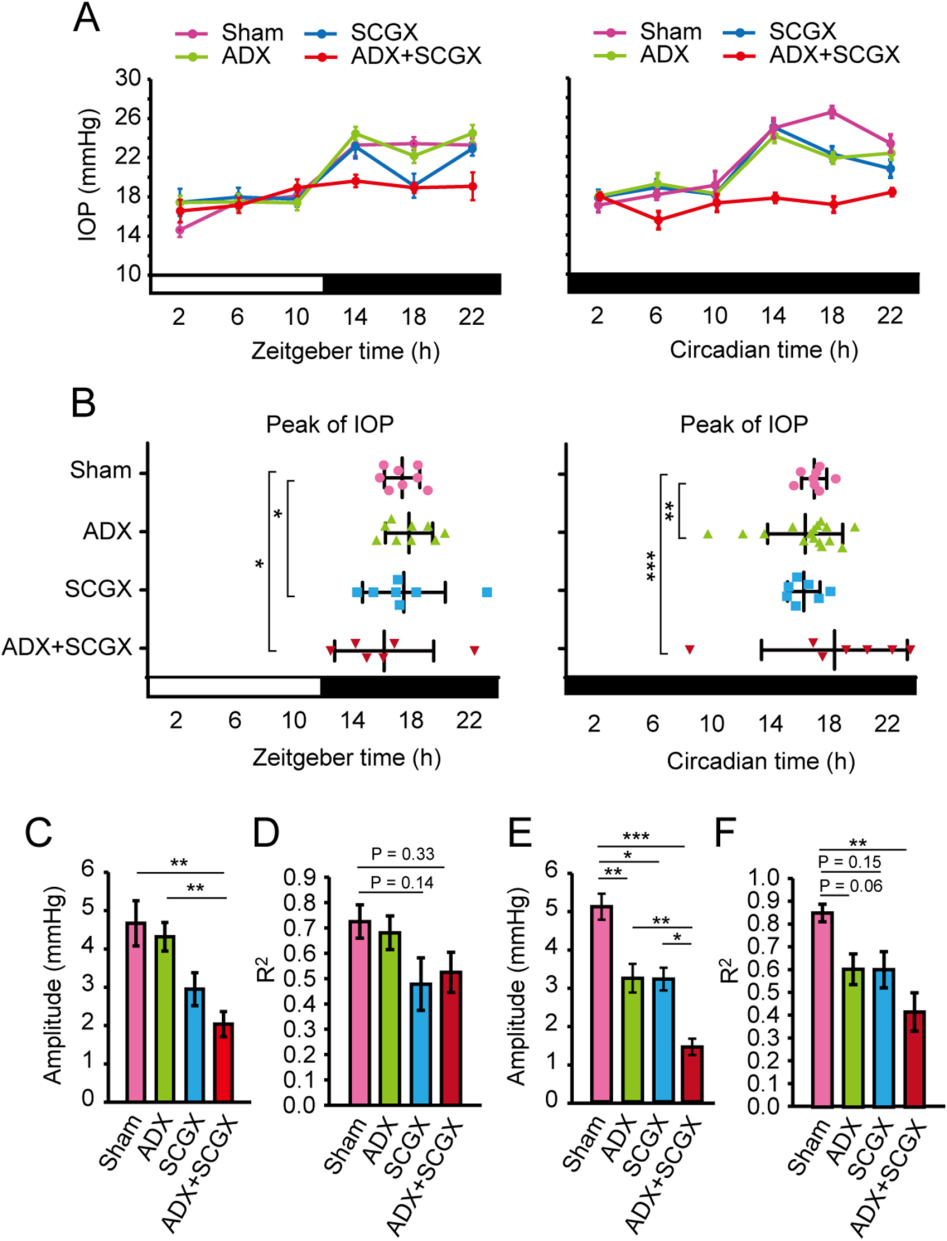
Effects of ADX and SCGX on temporal IOP rhythm in mice. (**A**) Temporal IOP profiles and (**B**) the plot of the peak phase determined using cosinor curve fitting in bilateral adrenalectomized (ADX) (*green*), superior cervical ganglionectomized (SCGX) (*blue*), and ADX+SCGX (*red*) mice kept under 12L12D (*left*) and DD (*right*) conditions. The time of light ON defines Zeitgeber time 0 (0800). CT based on the internal free-running period. The onset of activity in mice defines CT12 (2000). The rhythmic IOP changes (two-way repeated measure ANOVA, ***P < 0.0001 in sham mice, ADX, and SCGX mice [12L12D and DD]) were disrupted by ADX+SCGX (*P* < 0.05 [12L12D] and *P* > 0.05 [DD]). ADX and SCGX caused dispersion of the IOP rhythm peaks in 12L12D and DD (f-test, *F < 0.05, **F < 0.01, ***F < 0.001 vs sham mice). See also Supplementary Figure S1, and Supplementary Figure S2. *White* and *black bars* indicate light and dark phases, respectively. Mean ± SEM, *n* = 7 to 18. (**C, E**) Decreased amplitude and (**D, F**) correlation coefficient (R^2^) of rhythmicity was observed in (**C, D**) 12L12D and (**E, F**) DD conditions. Mean ± SEM, *n* = 7 to 18, *P < 0.05, **P < 0.01, ***P < 0.001 (one-way ANOVA, Tukey's multiple comparison test).

To address the effects of glucocorticoids and sympathetic pathways on IOP rhythm, we performed time course IOP measurements under 12L12D in the mice after bilateral ADX and SCGX. Initially, a normal circadian locomotor activity rhythm under 12L12D and DD conditions was performed in all mice (Supplementary Fig. S1A) and completely bilateral ADX mice (Supplementary Fig. S1B). Sham mice showed a clear IOP rhythm: lower in the day and higher at night (*P* < 0.001) (Fig. 1A). ADX affected IOP at some time points (*P* < 0.05 at zeitgeber time 18 [0200]), but IOP rhythm was retained (Fig. 1A, Supplementary Fig. S1C). SCGX also had a minor effect on the IOP rhythm (Fig. 1A, Supplementary Fig. S1C). In contrast, the dispersion of IOP rhythm peaks in ADX or SCGX mice varied between the 12L12D and the DD conditions (F < 0.05) (Fig. 1B, Supplementary Fig. S1D).

We next performed both ADX and SCGX in mice to confirm this relationship. This suppressed nocturnal IOP increase under the 12L12D condition, but yielded few rhythm changes (*P* = 0.05) (Fig. 1A, Supplementary Fig. S1C). We found similar results under the DD condition (*P* > 0.05, ADX+SCGX) (Fig. 1A, Supplementary Fig. S1C). We could not detect differences in IOP rhythm between 12L12D and DD except in the sham group (*P* > 0.05 for ADX, SCGX, and ADX+SCGX, *P* < 0.05 for the sham group) (Supplementary Fig. S1E). ADX and SCGX also dispersed the IOP rhythm peaks in both conditions (Fig. 1B, Supplementary Fig. S1D). A decreased amplitude and correlation coefficient (R^2^) of rhythmicity was observed under both 12L12D (Figs. 1C, 1D) and DD (Figs. 1E, 1F) conditions. In each mouse, circadian changes in IOP were arrested (Supplementary Fig. S2A–S2D). These results suggest that the transmission pathway of IOP rhythm is composed of two components and involves adrenal glucocorticoids and the sympathetic nervous system.

### CORT and NE Modulate IOP Rhythmicity

**Figure 2.**
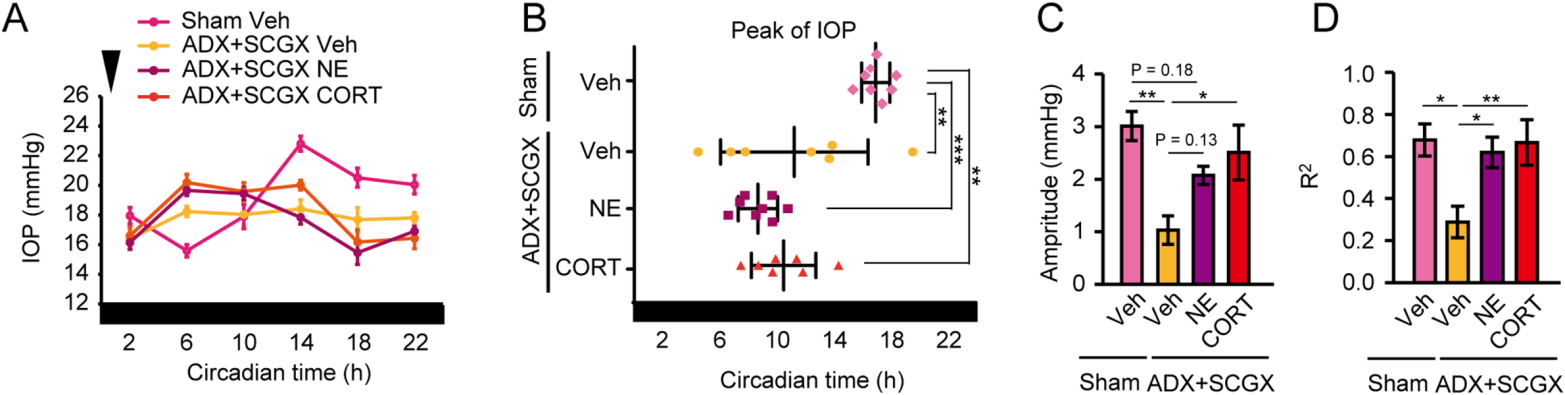
Ocular administration of CORT or NE generates IOP rhythm. Effects of NE (1 mg/mL) and CORT (1 mg/mL) instillation to bilateral eyes at CT0 to CT1 (*arrowhead*) on IOP rhythms of ADX+SCGX mice. (**A**) Mean and (**B**) a plot of the peak phase on cosinor curve fitting of the IOP rhythm. Administration of each of these induced significant antiphasic rhythm of IOP (two-way ANOVA, *P* < 0.0001 [sham (Veh), ADX+SCGX (CORT, NE)], *P* > 0.05 [ADX+SCGX (Veh)]). *Black bars* indicate the dark phases. Mean ± SEM, *n* = 7 to 8, **P < 0.01, ***P < 0.001 (one-way ANOVA, Tukey's multiple comparison test). (**C**) Decreased amplitude and (**D**) R^2^ were rescued by NE and CORT. Mean ± SEM, *n* = 7 to 8, *P < 0.05, **P < 0.01 (one-way ANOVA, Tukey's multiple comparison test).

To investigate the modulation of IOP rhythm by CORT and NE, we administered eyedrops containing CORT and NE, respectively, into bilateral eyes of ADX+SCGX mice, at 0 to 1, that is, during the physiologic antiphase, on the second day of DD. Administration of CORT and NE induced a weak but significant antiphase peak in IOP rhythm, as compared with the arrhythmicity of the control group (*P* < 0.0001 for sham [Veh], and ADX+SCGX [CORT, NE]; *P* > 0.05 for ADX+SCGX [Veh]) (Fig. 2A, Supplementary Fig. S3A). Moreover, the dispersed phase caused by ADX+SCGX was phase advanced owing to morning NE and CORT instillation, when compared with sham mice (Fig. 2B). NE and CORT almost restored the decreased amplitude and R^2^ (but not completely) to normal levels (Figs. 2C, 2D). These data suggest that CORT and NE may be involved in circadian regulation of the IOP rhythm. In contrast, the administration of CORT and NE with the correct physiologic phase, CT12 to CT13, also increased IOP (Supplementary Fig. S3A). The peak after instillation and IOP amplification effects were not different from the CT0 to CT1 group (Supplementary Fig. S3B–S3D), indicating a null time-dependent response.

To confirm whether the IOP rhythm is an autonomous rhythm or a driven output, we measured the IOP rhythm until the second day after instillation. The CORT- and NE-induced IOP changes were not sustained until the second day (Supplementary Fig. S3E), suggesting that IOP rhythms may be a driven output, independent of circadian clock regulation, and not self-sustainable after a single stimulation. We next administered eyedrops containing CORT and NE, respectively, into the bilateral eyes of sham mice at CT0 to CT1. A slight daytime increase of IOP and decrease of amplitudes was detected; however, the nocturnal IOP increase was maintained and the phases did not shift (Supplementary Figs. S3G–S3J). These findings indicate that the IOP rhythm is perhaps purely a systemic-driven response.

### Glucocorticoid Receptors and β2-Adrenergic Receptors are Expressed in the Ciliary Body

**Figure 3.**
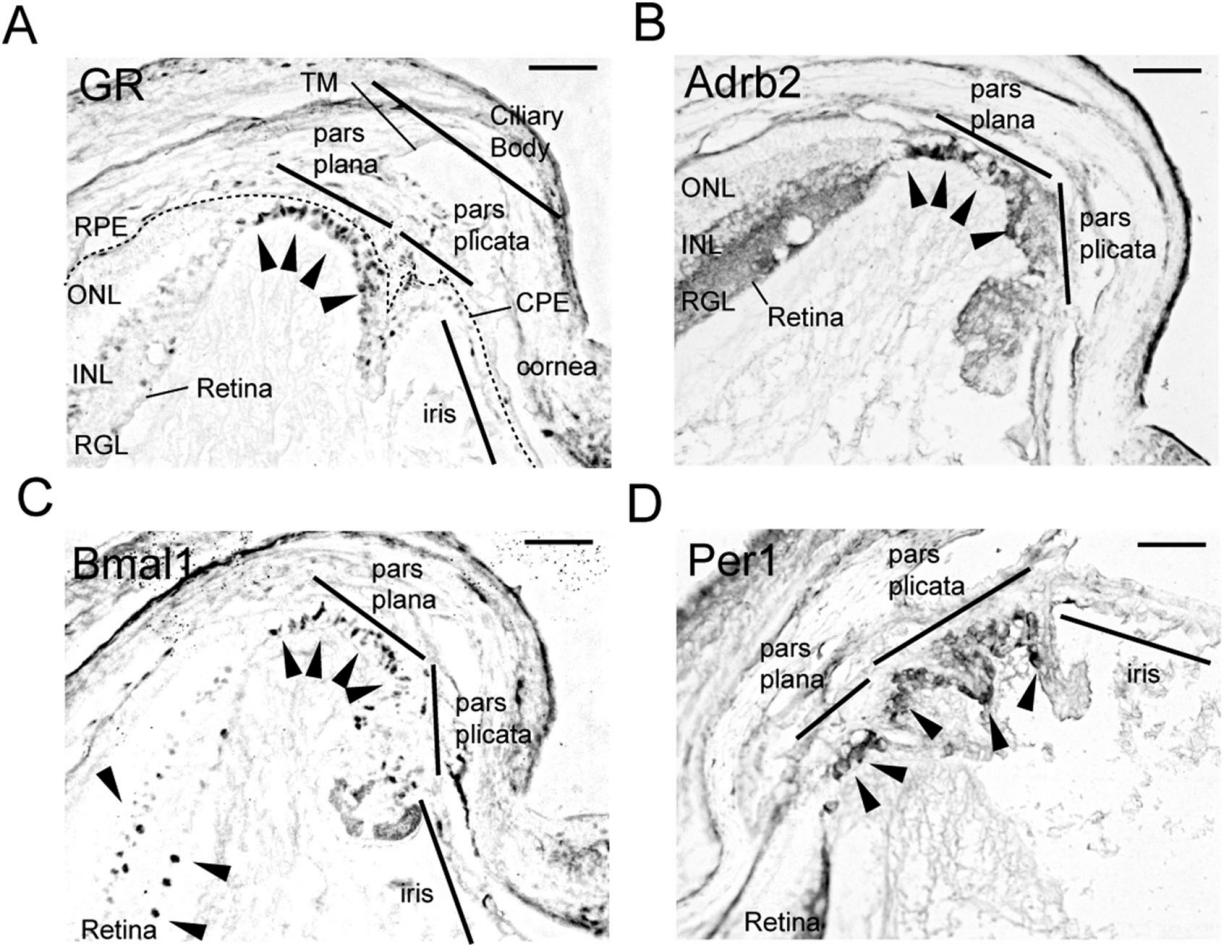
Localizations of GR, Adrb2, and Bmal1 in the ciliary body. Representative images of (**A**) GR, (**B**) adrenergic β2-receptors (Adrb2), (**C**) Bmal1, and (**D**) Per1 immunoreactivity in the retina and ciliary body of albino Balb/c mice by immunohistochemistry. Strong immunoreactivity (*arrowhead*) was seen in the pars plana of the ciliary body. Scale bar: 100 µm. CPE, ciliary pigmented epithelium; INL, inner nuclear layer; ONL, outer nuclear layer; RGL, retinal ganglion cell layer; TM, trabecular meshwork.

As the expression of β2-adrenergic receptors (Adrb2) in the nonpigmented epithelium (NPE) of the human ciliary body is higher than that of β1-adrenergic receptors,^29^ and because GR are also expressed in human^30^ and chicken ciliary bodies,^31^ we sought to confirm the localizations of expression for GR and Adrb2. Immunohistochemistry revealed strong expression of GR and Adrb2 in the pars plana of the ciliary NPE and weak expression in the retinal ganglion cell layer, inner nuclear layer, and pars plicata of the ciliary NPE, of albino Balb/c (Figs. 3A, 3B) and C57BL/6J mice (Supplementary Fig. S4A). The circadian clock protein Bmal1 and Per1 were identified in the NPE, but not in the trabecular meshwork in Balb/c mice (Figs. 3C, 3D) and C57BL/6J mice (Supplementary Fig. S4B). These findings indicate that CORT and NE act on the ciliary body and regulate IOP rhythm with a nocturnal increase by mediating the production of aqueous humor.

### CORT and the Sympathetic Pathway Regulate Ciliary Circadian Rhythm

**Figure 4.**
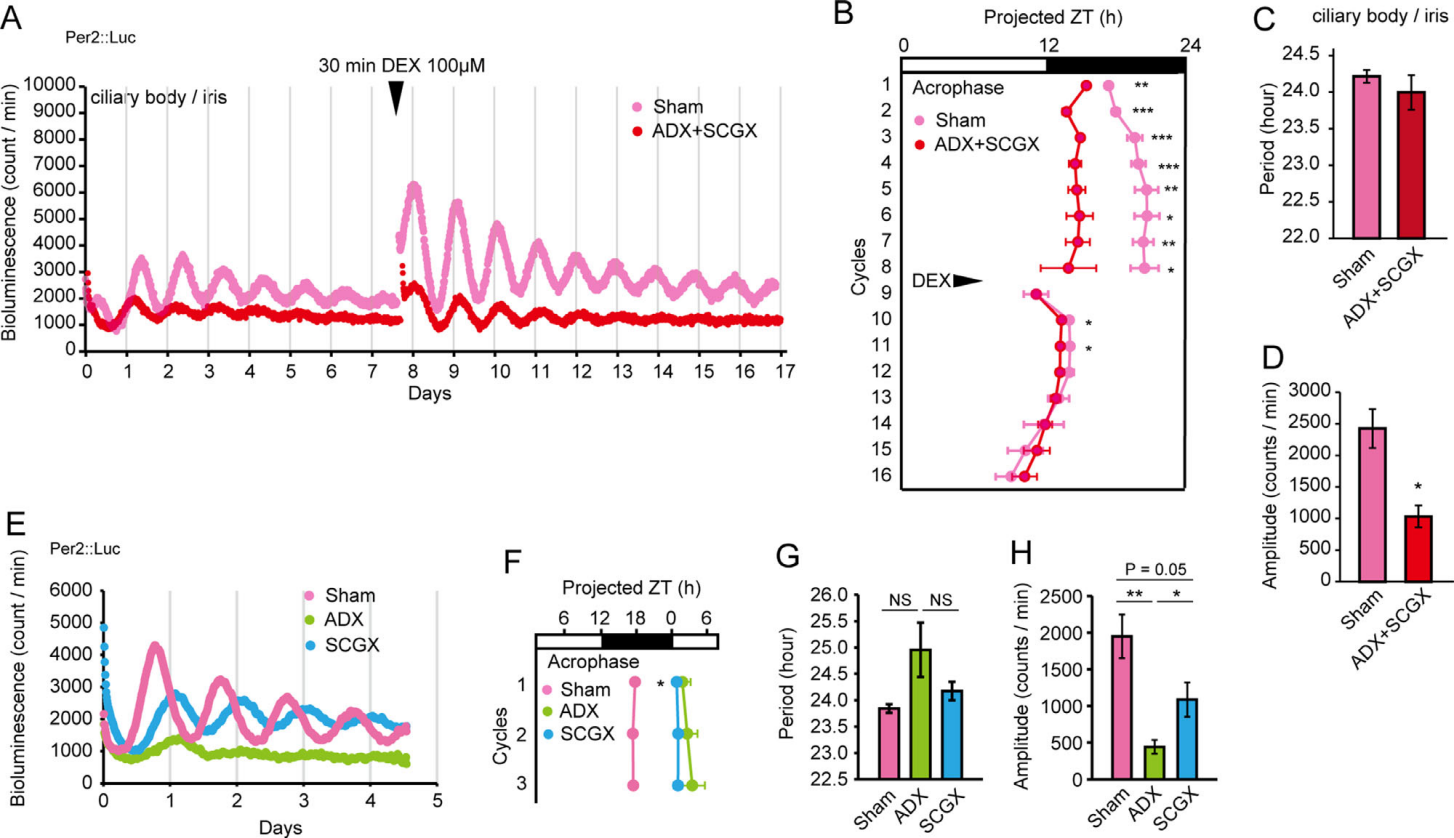
Effect of ADX and SCGX on Per2::Luc rhythm in the ciliary body. (**A**) Bioluminescence rhythms of Period 2::luciferase (Per2::Luc) and effect of the glucocorticoid receptor agonist DEX (100 nM for 30 minutes) on phase shifting in the cultured ciliary body/iris of sham and ADX/SCGX mice, and (**B**) those acrophase profiles. *White* and *black bars* indicate the light and dark phases, respectively. Means ± SEM, *n* = 4. **P < 0.01, ***P < 0.001 (two-way repeated measure ANOVA with Tukey's multiple comparison test). (**C, D**) ADX and SCGX dampened (**C**) and phase shifted (**D**) the Per2::Luc rhythm in the ciliary body/iris. (**E**) Bioluminescence rhythms of Per2::Luc in the cultured ciliary body/iris of sham, ADX, and SCGX mice, and (**F**) the acrophase profiles. *White* and *black bars* indicate the light and dark phases, respectively. Means ± SEM, *n* = 4–5,*P < 0.05 (two-way repeated measure ANOVA with Tukey's multiple comparison test, *P* < 0.0001 vs sham). (**G**) Period and (**H**) amplitude of the first cycle in Per2::Luc rhythm. ADX and SCGX dampened the Per2::Luc rhythm in the ciliary body/iris. Means ± SEM, *n* = 4 to 5, *P < 0.05, **P < 0.01 (one-way ANOVA, Tukey's multiple comparison test). ZT, zeitgeber time.

To understand the effect of ADX and SCGX on circadian clock rhythm in the ciliary body, we next performed real-time monitoring of Per2::Luc in a culture of the iris/ciliary body and SCN slice from ADX and SCGX Per2::Luc knock-in mice. As expected, the bioluminescence rhythm was markedly damped and phase-advanced by approximately 4 hours in the iris/ciliary body culture (Figs. 4A, 4B), whereas no differences were observed in the SCN slice cultures (Supplementary Fig. S5). Although ADX and SCGX did not affect the periodicity of rhythm in the iris/ciliary body culture (Fig. 4C), its significantly decreased amplitude (Fig. 4D), indicating circadian rhythm disruption in the iris/ciliary body by ADX and SCGX. Furthermore, Per2::Luc rhythm in the cultured iris/ciliary body could be induced and entrained by DEX administration (Figs. 4A, 4B). The rhythms, even those in ADX and SCGX mice, were self-sustaining after a single DEX stimulation, in contrast with the IOP rhythm after CORT instillation (Fig. 2A), indicating independent regulation of the IOP rhythm by the local clock. Furthermore, when we examined the individual effects of ADX and SCGX on the ciliary clock, ADX markedly attenuated Per2::Luc rhythm and resulted in a phase delay of approximately 8 hours, whereas SCGX slightly damped the rhythm and resulted in a marked phase delay of approximately 7 hours (Figs. 4E–4H). ADX or SCGX alone did not affect the period (Fig. 4G). It is possible that CORT and NE act additively on the ciliary body to generate rhythmicity and achieve an appropriate phase of IOP rhythm; however, mediation by ciliary clock remains unclear.

### The Ciliary Clock does not Regulate IOP Rhythm

**Figure 5.**
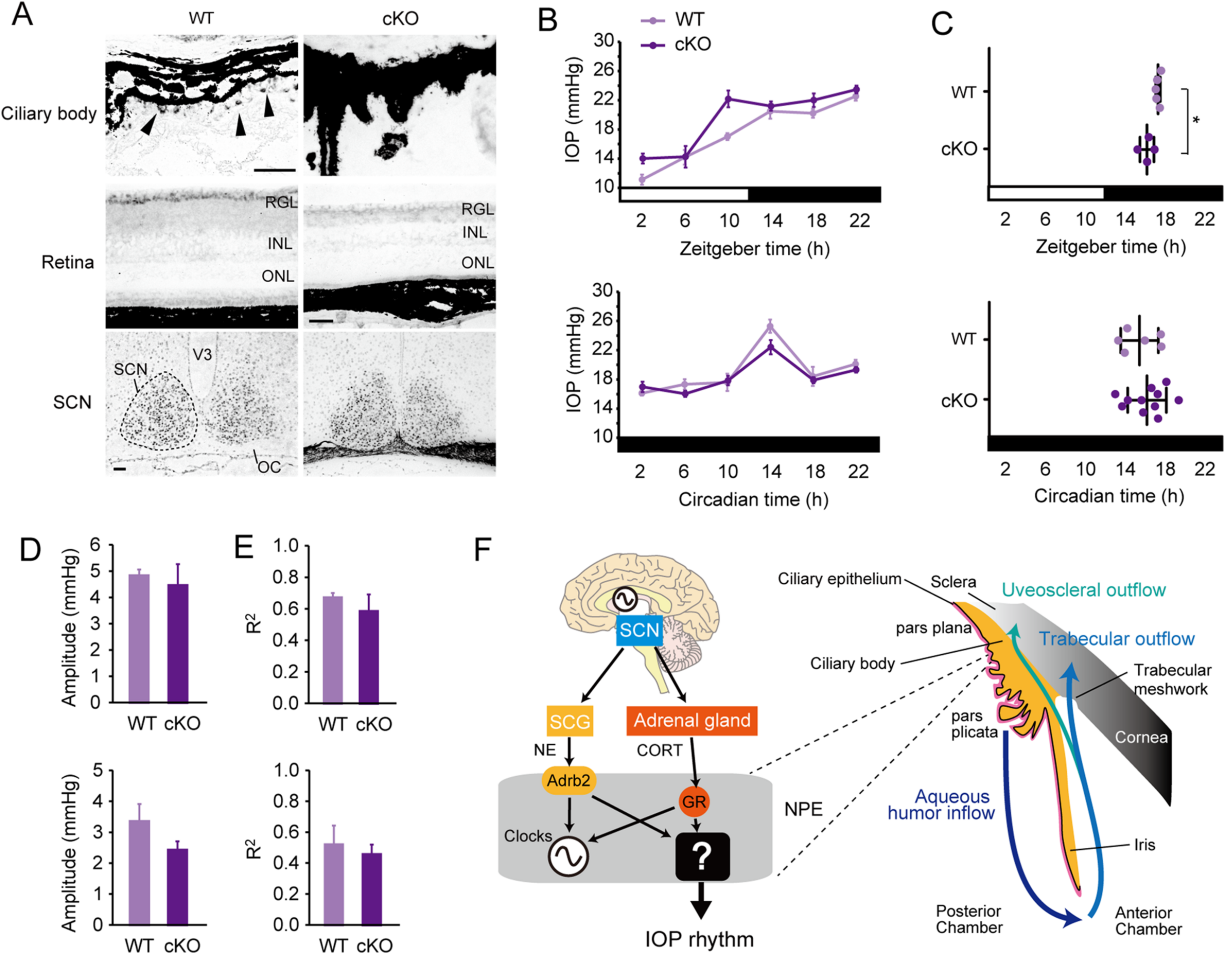
Topical clock disruption does not affect IOP rhythm. (**A**) Immunohistochemistry of Bmal1 in the ciliary body, retina, and the SCN of Chx10–Cre-negative (wild-type [WT]) and Cre-positive retina–ciliary epithelium-specific *Bmal1*-knockout (cKO) mice. Bmal1 expression was disrupted specifically in the retina and ciliary body. Scale bar: 50 µm. (**B**) Temporal changes of IOP in WT and cKO mice under 12L12D and DD conditions. IOPs were slightly increased only under 12L12D condition, but the rhythmicity was maintained in cKO mice (two-way repeated measure ANOVA, *P* < 0.05 [12L12D], *P* > 0.05 [DD] vs. WT). White and black bars indicate light and dark phases, respectively. Means ± SEM, *n* = 4 to 6 (12L12D), *n* = 6 to 11 (DD). (**C**) The plot of the peak phase determined using cosinor curve fitting in WT and cKO mice kept under 12L12D (*top*) and DD (*bottom*) conditions. *P < 0.05 (*t*-test), F > 0.05 (f-test). (**D**) Amplitude and (**E**) R^2^ of rhythmicity were normal. *P* > 0.05 (*t*-test). (**F**) Regulatory pathways of IOP rhythm by the SCN through the adrenal glucocorticoid and sympathetic nerve pathway and an anatomical model. Circadian timing signals of NE and CORT are mainly transmitted to the ciliary epithelium (pars plana and pars plicata) for aqueous humor production, which seems to be independent of the ciliary clock. The aqueous humor is drained by two pathways, namely a trabecular outflow and an uveoscleral outflow. The molecular mechanism of circadian regulation in aqueous humor producing and drainage remains unknown. INL, inner nuclear layer; OC, optic chiasma; ONL, outer nuclear layer; RGL, retinal ganglion cell layer; V3, third ventricle.

To identify the role of the ciliary clock in IOP rhythm generation, we generated retina-ciliary epithelium-specific *Bmal1* knockout mice (cKO) by crossing *Bmal1* flox (wild type) and *Chx10-Cre* mice (Supplementary Fig. S6A, S6B). In cKO mice, Bmal1 expression decreased in the ciliary body NPE, retinal ganglion cells, and the inner nuclear layer of the retina, but was retained in the SCN (Fig. 5A). cKO mice also maintained normal circadian rhythmicity of locomotor activity under 12L12D and DD conditions (Supplementary Fig. S6C, S6D). These findings indicate a tissue-specific loss of Bmal1 function. However, the IOP rhythm of cKO mice showed almost no difference from the wild-type mice (two-way ANOVA, *P* > 0.05 vs. wild-type) (Fig. 5B). Phases, amplitudes, and R^2^ were also almost normal (Figs. 5C–5E). These findings suggest that the IOP rhythm is directly driven by CORT or NE, but not via the ciliary clock. However, the temporal patterns of IOP rhythm were slightly different between the 12L12D and DD conditions (Fig. 5B). The ciliary clock may, therefore, be necessary for achieving appropriate IOP rhythm through light stimuli.

## Discussion

Although ADX and SCGX alone maintained IOP rhythmicity,^13^^,^^24^ adrenal glucocorticoid and sympathetic NE alone could not fully account for the mechanisms underlying the IOP rhythm.^7^^,^^19^ We found that dual pathways of CORT and sympathetic nervous system can drive the rhythm (Fig. 5F). We also observed the expression of CORT and NE receptors in the ciliary body NPE (Fig. 3), consistent with previous reports,^29^^,^^31^ where clock proteins are also expressed (Fig. 3).^32^ Also, CORT and NE act additively on the ciliary body to generate rhythmicity and a phase reset (Fig. 4).^18^ In fact, DEX administration causes a phase-shift within the bone.^33^ ADX along with NE receptor blockers induces a phase delay in Per2::Luc expression in the kidney and liver.^34^ Although the circadian clock regulates the IOP rhythm,^35^ its mediation by the ciliary clock is unclear. In the present study, since cKO mice (Supplementary Fig. S6)^27^ were maintained with IOP rhythmicity (Fig. 5), we conclude that IOP rhythm is directly driven by either CORT or NE but not by an autonomous rhythm via the ciliary clock (Fig. 5F). This clock-independent regulatory pathway is consistent with the circadian regulation of the pineal gland, periventricular organ like the eye.^36^ The circadian rhythms of osteoclast-related genes are also regulated by CORT, independent of the local clock.^37^

The physiologic implications of the dual regulatory pathways remain unknown. However, because CORT levels are increased through diet,^38^ a sympathetic nervous pathway may act as a backup. In fact, both β-adrenergic signaling and glucocorticoids are mediators of circadian rhythm output from the SCN to osteoblasts.^20^ The CORT and sympathetic pathways may also interact, because NE or CORT instillation alone could not fully generate the amplitude of IOP rhythm (Fig. 2). SCGX in rats suppresses the circadian CORT rhythm.^39^ Adrb2 can regulate GR transactivation.^22^ In contrast, although CORT promotes the expression of several genes through glucocorticoid-response elements,^40^ it modulates Adrb2 expression through the promotor glucocorticoid-response elements.^21^ Furthermore, ADX combined with NE receptor blockers completely blocks exercise-induced entrainment of locomotor activity.^34^ Therefore, the interaction of CORT and NE could generate an appropriate IOP rhythm.

Although glucocorticoid secretion peaks at the light offset,^41^ and NE is released with a nocturnal peak from the SCG in rodents,^42^ these rhythms are antiphasic in nocturnal and diurnal animals.^41^^–^^44^ IOP is elevated at night in nocturnal mice (Fig. 1),^7^^,^^12^ diurnal humans, and monkeys.^6^^,^^9^^,^^45^ However, the phase of IOP rhythm seems to be different between the nocturnal and diurnal animals. In nocturnal animals (mice, rabbit, and cat), the IOP rhythm peaks early at night (Fig. 1).^7^^,^^46^ In healthy humans, although IOP changes rapidly depending on various conditions, it seems to be elevated during night and peaks from the late night to early morning.^6^^,^^9^^,^^47^ Such differences may be explained by oppositely phased rhythmed SCN rhythm outputs between the species.

CORT and NE receptors are expressed more strongly and densely in the pars plana compared with the pars plicata (Fig. 3, Fig. 5F). Because the IOP rhythm seems to be directly driven by CORT or NE, some genes that respond directly to CORT and NE may be involved. Sympathetic nerve pathways connect to regulate aqueous humor production^48^ through the expression of some genes, including the gene that codes carbonic anhydrase.^49^ The NPE in the pars plicata and pigmented epithelium cells in the pars plana appear to contain carbonic anhydrase.^50^ Carbonic anhydrase II is colocalized with GRs in mouse brain,^51^ and DEX stimulation increases carbonic anhydrase II expression. Thus, it seems plausible that the pars plana itself can produce an aqueous humor rhythm. In contrast, since the pars plicata is larger in area compared with the pars plana, and the NPE of the pars plana and pars plicata are linked via tight gap junctions, electronic rhythmic signals may be transmitted from the pars plana to the pars plicata through these tight junctions.

Aqueous humor outflow components may be also involved in IOP circadian rhythm.^52^ Given the camera-like function of the eye, a higher IOP would be better for focusing. However, because this is inextricably linked to cell death, a mechanism for decreasing IOP is also essential (Fig. 5F). The ras homolog gene family (Rho) is a member of small GTPases associated with the cytoskeleton. Although Rho kinase inhibitors decrease the IOP by increasing the drainage of aqueous humor into Schlemm's canal, inhibition of Rho member A (RhoA) prevents nocturnal IOP elevation.^53^ Schlemm's canal drainage has been reported to be inhibited by CORT, which prevents enzyme release by stabilizing the lysosomal membranes, which can lead to outflow obstructions that cause glaucoma.^54^ Glucocorticoids can increase IOP in some individuals, and several patients with POAG exhibit high sensitivity to glucocorticoids. However, in our study, GR signals were weak in the trabecular meshwork and Schlemm's canal but were detected in the ciliary body NPE (Fig. 3), consistent with previous reports.^31^ Local steroid administration induces nuclear transfer of GR in the iris/ciliary body of rabbits.^55^ This may involve molecular interactions with the trabecular meshwork and increase the resistance of drainage. In contrast, although the IOP is increased and aqueous humor production is decreased at night, the trabecular outflow is not altered significantly.^56^ This paradox may be explained by the episcleral venous pressure and the IOP-independent uveoscleral outflow (ciliary muscle), other important determinants of IOP (Fig. 5F). Particularly, 30% to 42% of aqueous humor leaves the eye through the uveoscleral pathway in several young mice strains.^57^ In different species, it is estimated that it carries 3% to 82% of the total aqueous humor outflow.^58^ In the present study, GR and Adrb2 were expressed weakly in the ciliary muscle (Fig. 3). Thus, circadian IOP may be influenced by the presence of local clock genes in this outflow apparatus instead of the ciliary NPE. The mechanism of circadian drainage needs further elucidation.

In this study, we addressed the effect of SCGX and ADX on the ciliary body clock and rhythm. Because the evaluated rhythms were affected in explants, these effects may be caused by explant damages and not be just a matter of lack of synchronizing inputs. Real-time monitoring of ciliary body rhythm in vivo will elucidate real effects. Furthermore, we demonstrate success, for the first time, in eliminating the IOP rhythm completely. However, we do not fully understand the underlying mechanism of impaired entrainment in ADX and SCGX mice. Although we report NPE clock-independent regulation of IOP rhythm, the effects of the local clock in the outflow apparatus of aqueous humor on the rhythm remains unclear. Latanoprost, a prostaglandin analog, decreases IOP by increasing the uveoscleral outflow of the aqueous humor in a time-dependent manner.^59^^,^^60^ Further studies on the interactions between CORT and NE, and the circadian molecular mechanisms could help to devise a time-dependent therapy for glaucoma.

## Supplementary Material

Supplement 1
