# Supplementary material for: Circadian Regulation of IOP Rhythm by Dual Pathways of Glucocorticoids and the Sympathetic Nervous System
Source: Invest Ophthalmol Vis Sci. 2020 Mar 17;61(3):26. doi: 10.1167/iovs.61.3.26 (PMC7401506; doi:10.1167/iovs.61.3.26)
Supplement: Supplement 1 [file iovs-61-3-26_s001.pdf]

Circadian regulation of IOP rhythm by dual pathways of glucocorticoids and sympathetic nervous system

Authors

Keisuke Ikegami[1*], Yasufumi Shigeyoshi[2], and Satoru Masubuchi[1]

Affiliations

[1] Department of Physiology, School of Medicine, Aichi Medical University, 1-1 Yazako-karimata, Nagakute, Aichi, 480-1195, Japan

[2] Department of Anatomy and Neurobiology, Faculty of Medicine, Kindai University, 377-2 Ohno-Higashi, Osaka-Sayama, Osaka 589-8511 Japan

*Correspondence:
Keisuke Ikegami, Ph.D.
Department of Physiology, School of Medicine
Aichi Medical University, 1-1 Yazako-karimata, Nagakute 480-1195 Japan
Phone +81 561 62 3311
Fax +81 561 63 1289
ikegami.keisuke.910@mail.aichi-med-u.ac.jp

Supplementary Information

Materials and Methods

Animals

For determining the locomotor activity rhythm, 8-week-old male C57BL/6J mice and cKO mice were individually housed in cages. Each cage was equipped with an infrared area motion sensor (human body detection sensor [PS-3241, Elekit, Fukuoka, Japan] or infrared human body sensor kit [NP-L, Sengoku-densho, Osaka, Japan] with a NaPiOn sensor [AMN32111, Panasonic, Osaka, Japan] [1]) that detects spontaneous movements of the animal in the cage. Data of locomotor activity was recorded on chronobiology kit (Bio Research Center, Nagoya, Japan) or a computer [1] and then analyzed with a Clock Lab (Actimetrics, Wilmette, IL). Activity was recorded in 1-min bins.

Measurement of CORT

We selected completely ADX mice by measuring serum corticosterone using a corticosterone ELISA kit (501320, Cayman Chemical, Ann Arbor, MI) (Fig. S1). Serum preparation was performed as in our previous report [2]. The IOP experiments on these mice were conducted at least 2 weeks after surgery.

References

1. Nagano M, Ikegami K, Minami Y, et al. Slow shift of dead zone after an abrupt shift of the light-dark cycle. *Brain Res*. 2019;1714:73-80. doi:10.1016/j.brainres.2019.02.014

2. Ikegami K, Liao XH, Hoshino Y, et al. Tissue-specific posttranslational modification allows functional targeting of thyrotropin. *Cell Rep*. 2014;9:801-809. doi:10.1016/j.celrep.2014.10.006

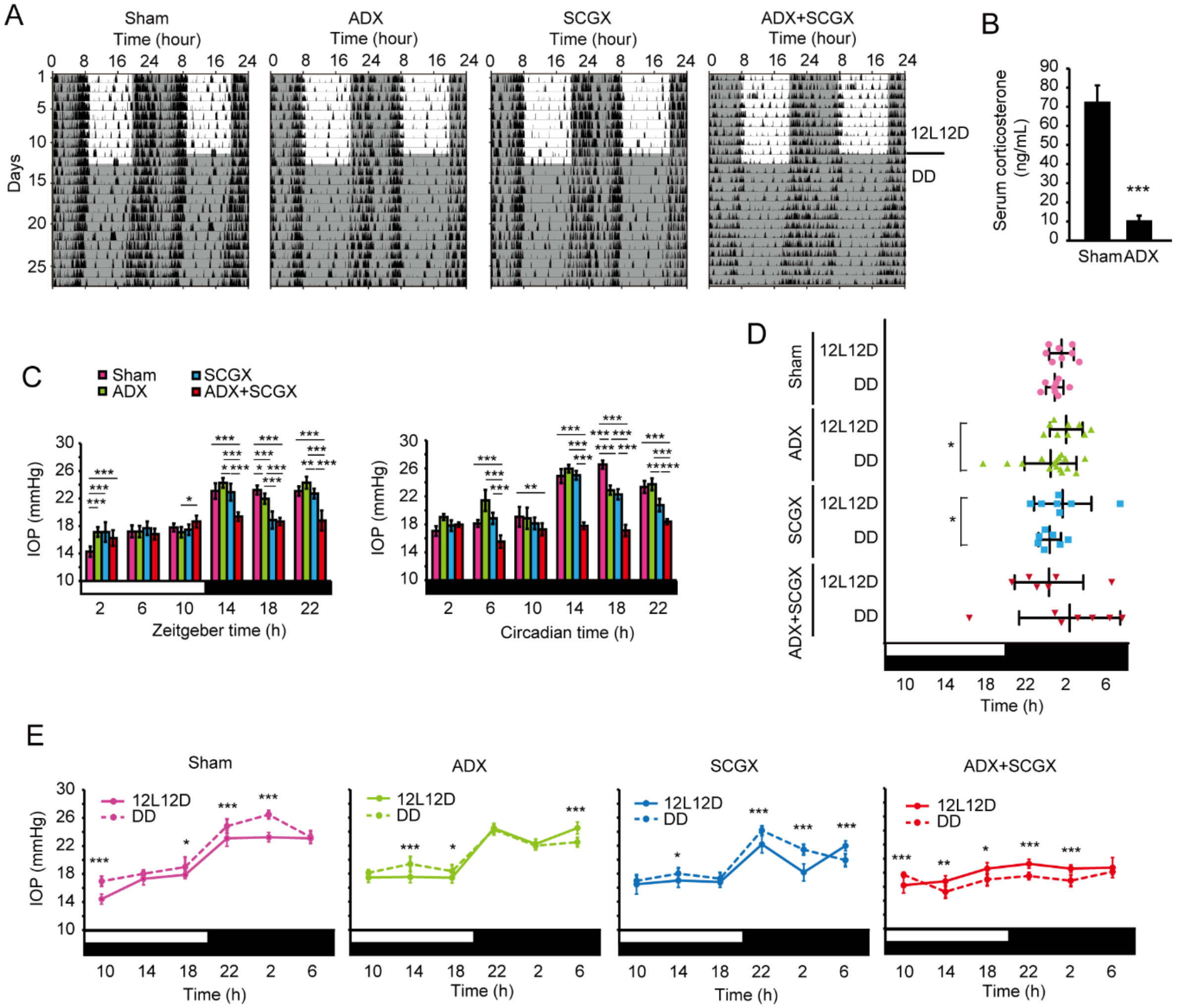

Fig. S1. Effects of adrenal glucocorticoid and sympathetic norepinephrine on temporal changes of IOP in mice. (**A**) Locomotor activity rhythm in bilateral adrenolectomized (ADX) and superior cervical ganglionectomized (SCGX) mice under 12-h:12-h light:dark cycles (12L12D) and constant dark (DD) conditions. The gray area of each graph indicates dark phases. (**B**) Effect of ADX on serum corticosterone concentration. Mean ± SEM, n = 8–9. ***p < 0.001 (*t*-test). (**C**) Effects of bilateral ADX (green), SCGX (blue), and ADX+SCGX (red) on intraocular pressure (IOP) profiles in mice kept under 12L12D (left) and DD (right) conditions. Mean ± SEM, n = 7–18, *p < 0.05, **p < 0.01, ***p < 0.001 (two-way repeated measure ANOVA, Tukey's multiple comparison test). (**D**) Dispersion of

IOP rhythm peaks in ADX or SCGX mice in 12L12D and DD conditions (f-test, *F < 0.05). (**E**) We could not detect differences in the IOP rhythm between 12L12D and DD except Sham group (two-way repeated measure ANOVA, p > 0.05 [ADX, SCGX, and ADX+SCGX], p < 0.05 [sham], Tukey's multiple comparison test [*p < 0.05, **p < 0.01, ***p < 0.001 12L12D vs DD]).

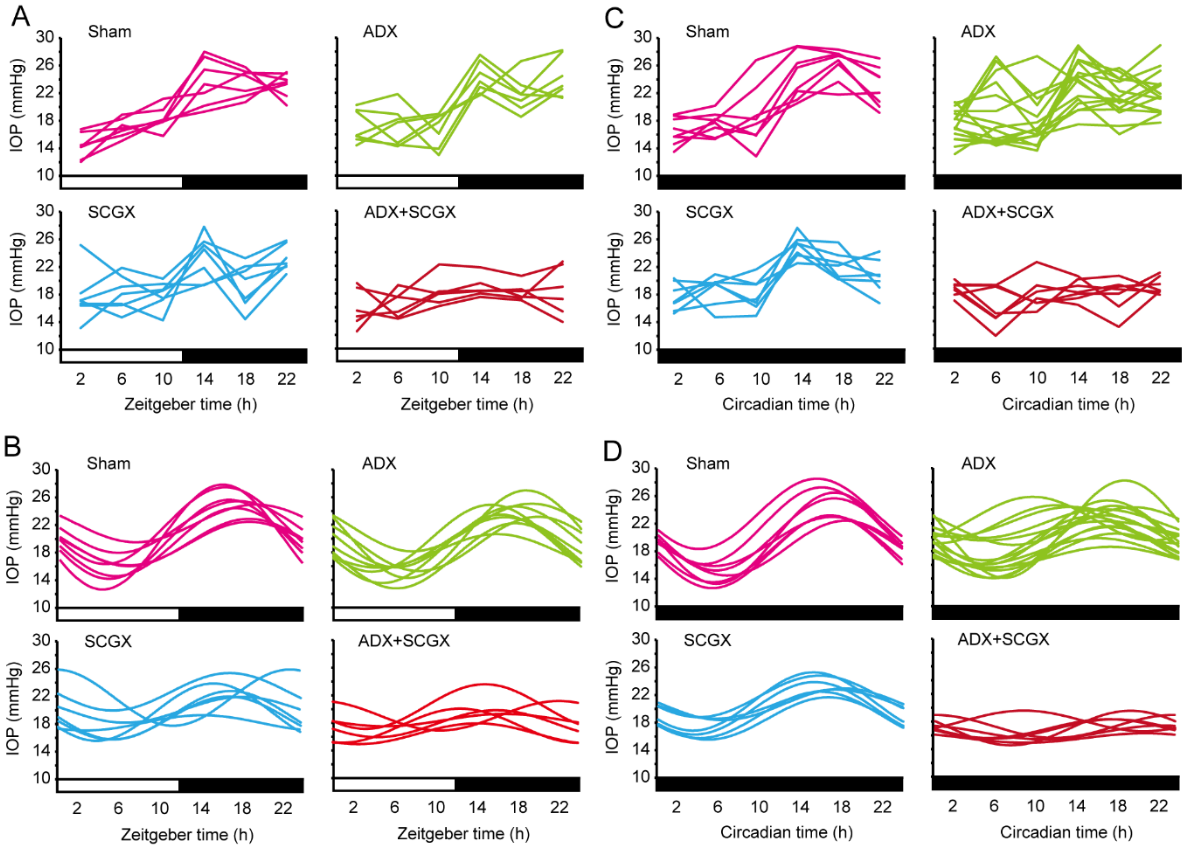

Fig. S2. Individual data of IOP rhythm.

Individual line graph and cosinor curve fitting of intraocular pressure (IOP) rhythm in sham, ADX, SCGX, and ADX+SCGX mice kept under 12L12D and DD conditions**.** n = 7–18.

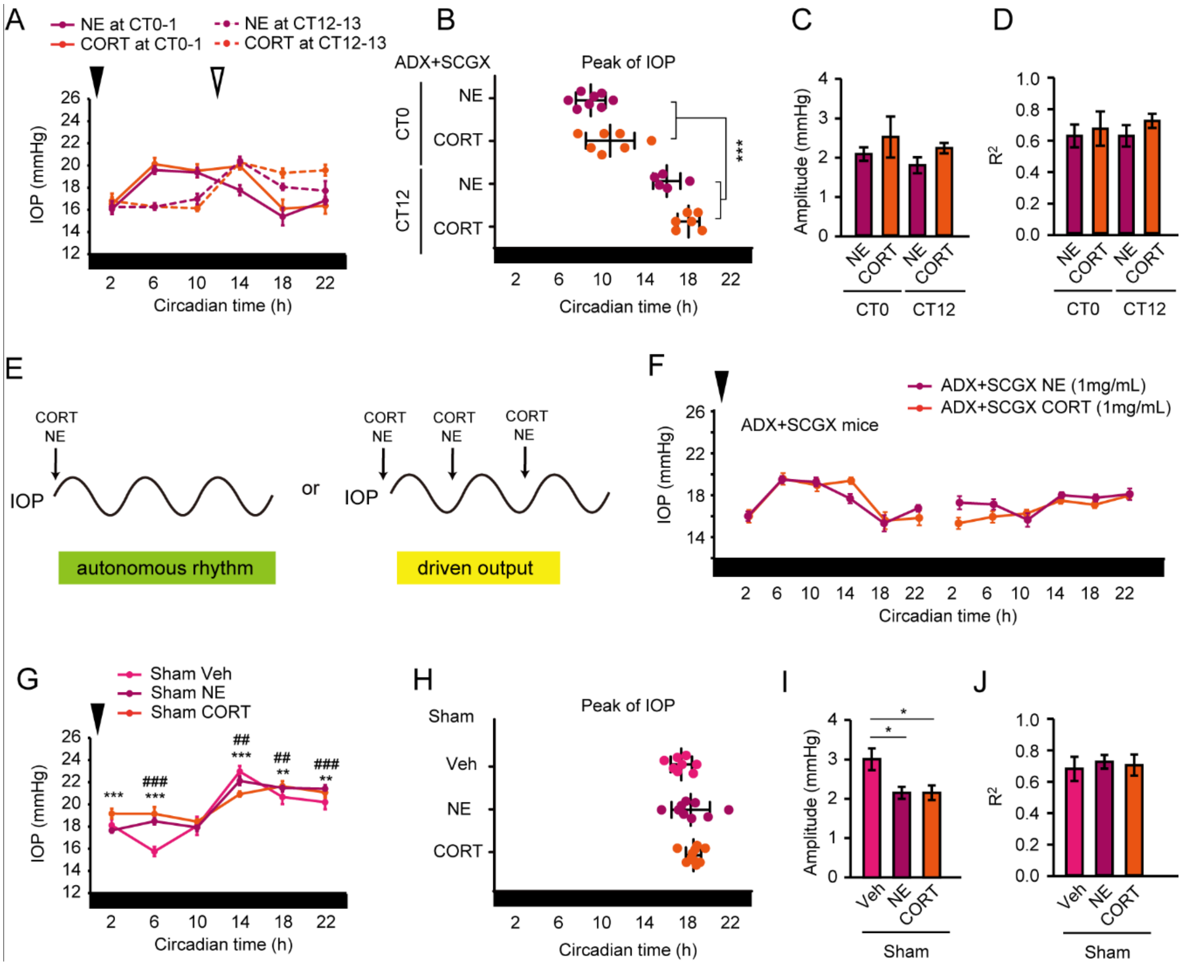

Fig. S3. Effects of ocular administration of NE and CORT on IOP rhythms

(**A, B**) Effects of NE and CORT instillation to bilateral eyes at circadian time (CT) 12 (2000) to CT13 (2100) (dot line and open arrowhead) on IOP rhythms of ADX+SCGX mice. (**A**) Mean and (**B**) a plot of the peak phase on the cosinor curve fitting of the IOP rhythm. Those data at CT0 to CT1 in Fig. 2 were replotted (solid line and closed arrowhead). Administration of each of these at CT12 to CT13 also induced a nocturnal increase of IOP (two-way ANOVA, $p < 0.001$). The peaks of IOP were delayed compared with those in mice injected at CT0 to CT1 (one-way ANOVA, Tukey's multiple comparison test, ***$p < 0.001$). Black bars indicate the dark phases. Mean ± SEM, n = 5–8. (**C**) Amplitude and (**D**) R^2 of the cosinor curve fitting of the IOP rhythm. Mean ± SEM, n =5–8, $p > 0.05$ (one-way ANOVA, Tukey's multiple comparison test). (**E**) The hypothesis of regulatory models in IOP rhythm. (**F**) Instillation of

NE and CORT induced the anti-phase of the diurnal IOP rhythm on the 1st day (replotted from Fig. 2), which disappeared on the 2nd day. (**G, H**) Effects of norepinephrine (NE, 1 mg/mL) and corticosterone (CORT, 1 mg/mL) instillation to bilateral eyes at CT 0 (0800) to CT1 (0900) (closed arrowhead) on IOP rhythms of Sham mice. (**G**) Mean and (**H**) a plot of the peak phase on the cosinor curve fitting of the IOP rhythm. Administration of each of these induced day-time increase of IOP, but the nocturnal increases of IOP were maintained (two-way ANOVA, $p > 0.05$ [ADX+SCGX (Veh)]). Mean ± SEM, n = 8–10, **$p < 0.01$, ***$p < 0.001$ vs Veh [CORT], $^{\#\#}p < 0.01$, $^{\#\#\#}p < 0.001$ vs Veh [NE] (two-way ANOVA, $p > 0.05$, Tukey's multiple comparison test). (**I**) Amplitude and (**J**) R^2 of the cosinor curve fitting of the IOP rhythm. Mean ± SEM, n = 8–10, *$p < 0.05$ (one-way ANOVA, Tukey's multiple comparison test).

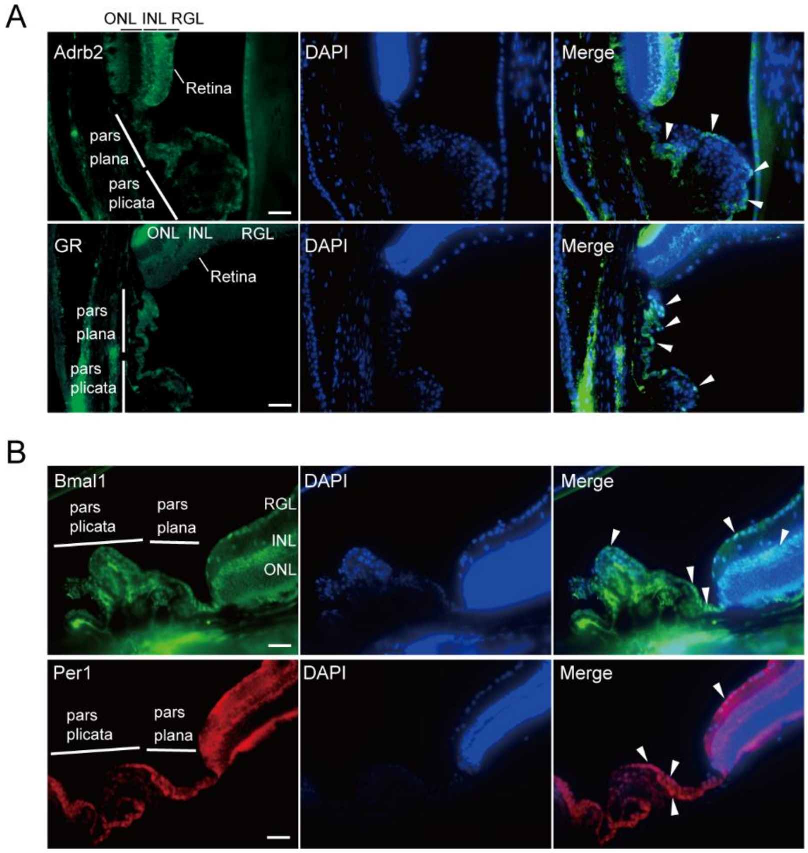

Fig. S4. Immunohistochemical analysis in the retina and ciliary body of C57BL/6J mice.

(**A**) Adrenergic β2-receptors (Adrb2) and glucocorticoid receptor (GR) immunoreactivity in the retina and ciliary body of C57BL/6J mice. (**B**) Immunoreactivity of clock protein Bmal1 and Per1 in the retina and ciliary body of C57BL/6J mice. Fluorescence immunohistochemistry revealed that immunoreactivity (white arrowhead) localized in the epithelia of the ciliary body. Scale bar: 100 μm. DAPI, 4',6-diamidino-2-phenylindole. RGL, retinal ganglion cell layer; INL, inner nuclear layer; ONL, outer nuclear layer.

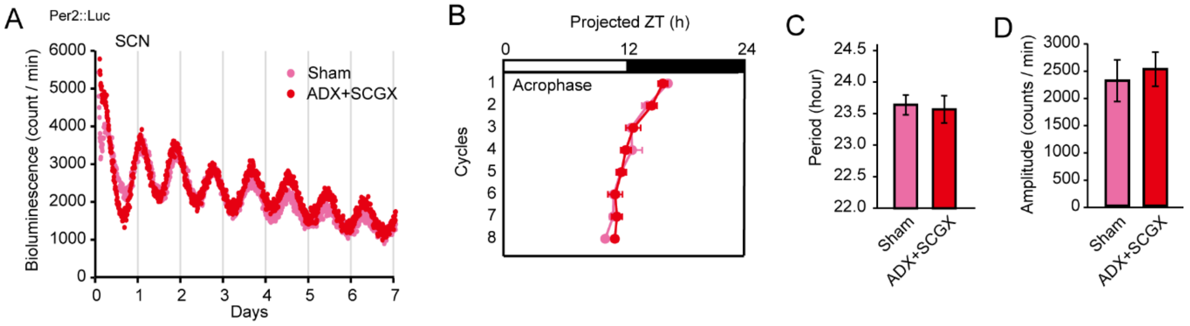

Fig. S5. Effects of ADX and SCGX on bioluminescence rhythms in the SCN.

(**A**) Bioluminescence rhythms of Per2::Luc in the cultured suprachiasmatic nucleus (SCN) slice of Sham and ADX/SCGX mice, and (**B**) the acrophase profiles. Means ± SEM, n = 4, p > 0.05 (*t*-test). ADX and SCGX did not affect the Per2::Luc rhythm in the SCN. White and black bars indicate the light and dark phases, respectively. The horizontal axis indicates the projected zeitgeber time (ZT). ZT0 (0800) is the light-on time in the housing room. (**C**) Period of the first three cycles and (**D**) amplitude of the first cycle in Per2::Luc rhythm. Means ± SEM, n = 4, p > 0.05 (*t*-test). ADX, adrenalectomy; SCGX, superior cervical ganglionectomy; Per2::Luc, Period 2::luciferase.

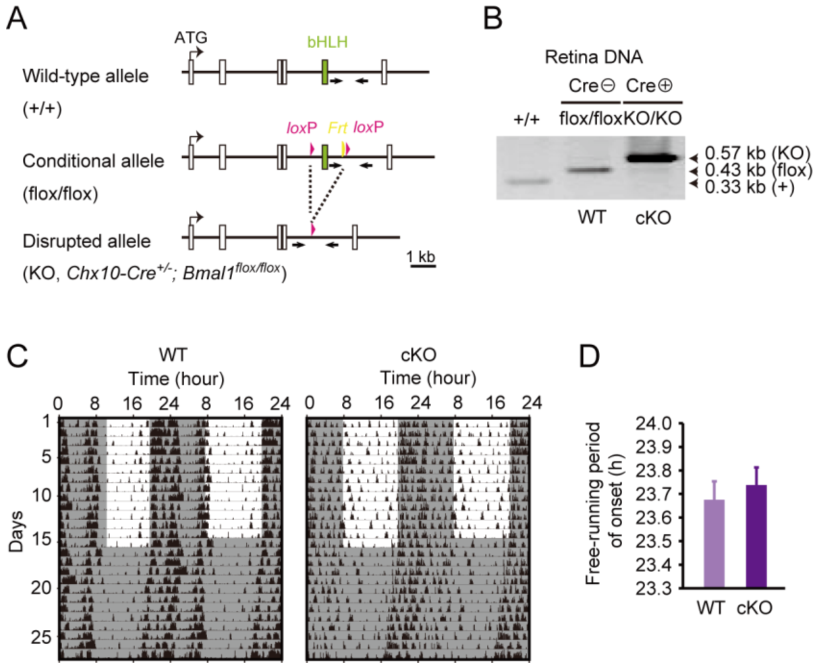

Fig. S6. Generation and locomotor activity rhythm of cKO mice.

(**A**) Targeting strategy and retinal-ciliary epithelium-specific conditional disruption of *Bmal1* using transgenic *Bmal1* $^{flox/flox}$ and *Chx10-Cre* mice. Opened boxes, exons; ATG, translation start site; bHLH, exon encoding basic helix-loop-helix domain (green); magenta triangles, loxP sites; yellow ovals, Frt sites. The arrows indicate the positions of PCR primers used for genotyping. (**B**) PCR products of 0.33, 0.43, or 0.57 kb were amplified from +/+, conditional allele (flox/flox, WT) or disrupted allele (KO/KO, cKO) of retina genome, respectively. (**C**) Representative double-plotted actogram of locomotor activity behavior of transgenic WT and cKO mice, and (**D**) their free-running period under the DD. Mice were kept under 12L12D for 2 weeks and then transferred to DD. The gray area indicates the dark phases. Means ± SEM, $p > 0.05$ (t-test), $n = 6$.
